# Osteochondral autologous transplantation for large steroid-induced osteonecrosis lesion of the knee in a young patient yielding satisfactory results despite only partial coverage: a case report

**DOI:** 10.1093/jscr/rjad487

**Published:** 2023-09-12

**Authors:** Shunsuke Akai, Tomoya Iseki, Ryo Kanto, Takuya Iseki, Shintaro Onishi, Yoshitaka Nakao, Shinichi Yoshiya, Toshiya Tachibana, Hiroshi Nakayama

**Affiliations:** Department of Orthopaedic Surgery, Hyogo Medical University, Nishinomiya, Hyogo 663-8501, Japan; Department of Orthopaedic Surgery, Hyogo Medical University, Nishinomiya, Hyogo 663-8501, Japan; Department of Orthopaedic Surgery, Hyogo Medical University, Nishinomiya, Hyogo 663-8501, Japan; Department of Orthopaedic Surgery, Hyogo Medical University, Nishinomiya, Hyogo 663-8501, Japan; Department of Orthopaedic Surgery, Nishinomiya Kaisei Hospital, Nishinomiya, Hyogo 662-0957, Japan; Department of Orthopaedic Surgery, Nishinomiya Kaisei Hospital, Nishinomiya, Hyogo 662-0957, Japan; Department of Orthopaedic Surgery, Nishinomiya Kaisei Hospital, Nishinomiya, Hyogo 662-0957, Japan; Department of Orthopaedic Surgery, Hyogo Medical University, Nishinomiya, Hyogo 663-8501, Japan; Department of Orthopaedic Surgery, Hyogo Medical University, Nishinomiya, Hyogo 663-8501, Japan

**Keywords:** steroid-induced osteonecrosis, osteochondral autograft transplantation, hyaline cartilage, osteochondral grafts, femoral condyle

## Abstract

Osteochondral autologous transplantation (OAT) is one of the most common surgical options for osteochondral disorders of the knee. In cases where OAT is performed for steroid-induced osteonecrosis, there are several problems potentially affecting the surgical outcomes such as large chondral damage area and compromised host bone. In addition, steroid administration for a long period of time may lead to extensive lesion, which poses difficulty in obtaining sufficient donor tissue. Those factors affect the prognosis of steroid-induced osteonecrosis resulting in inferior treatment outcomes. We present a young female with a large steroid-induced osteonecrosis lesion repaired only with two osteochondral plugs harvested from the healthy area. The reported case indicates that only partial osteochondral grafting limiting to the weight-bearing area may yield satisfactory outcome when OAT is performed for large steroid-induced osteonecrosis of the knee.

## Introduction

Systemic corticosteroid administration is the most common cause of secondary osteonecrosis of the knee in active young active patients with large osteochondral lesions [1]. In surgical treatment of osteochondral lesions, osteochondral autologous transplantation (OAT) is one of the popularly performed procedures and satisfactory surgical outcome has been generally reported [2–4]. However, in cases where OAT is performed for steroid-induced osteonecrosis, there are several problems that may affect the outcomes such as large chondral damage, compromised host bone, and difficulty in obtaining sufficient donor tissue. Consequently, clinical outcomes following OAT performed for steroid-induced osteonecrosis are generally inferior compared with other types of osteochondral lesions such as focal cartilage lesion and osteochondritis dissecans. In this report, we present a case in which a large steroid-induced osteonecrosis lesion was repaired using only two osteochondral grafts harvested from healthy area in the ipsilateral knee, as it was difficult to obtain a sufficient number of grafts because of bone necrosis in both knees.

## Case report

**Figure 1:**
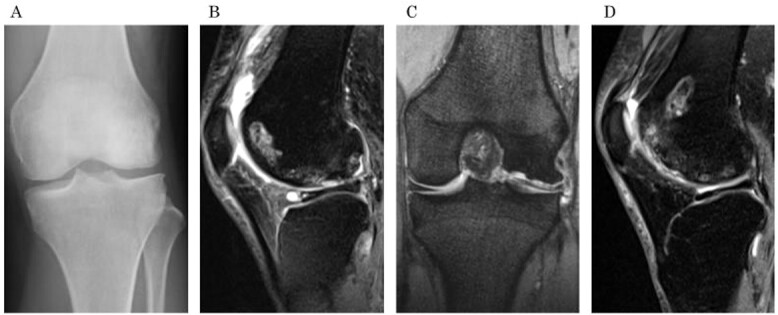
Preoperative radiographs and MRI. (a) An anteroposterior radiograph of the left knee shows the flattened lateral femoral condyle and irregular lateral joint surface. (b) A sagittal fat saturation T2-weighted image and (c) a coronal T2-weighted image show large osteonecrosis of the left lateral femoral condyle and lateral discoid meniscal tear associated with cyst formation. (d) A sagittal fat saturation T2-weighted image of right knee (contralateral knee) shows osteonecrosis of the lateral condyle similar to the left knee.

**Figure 2:**
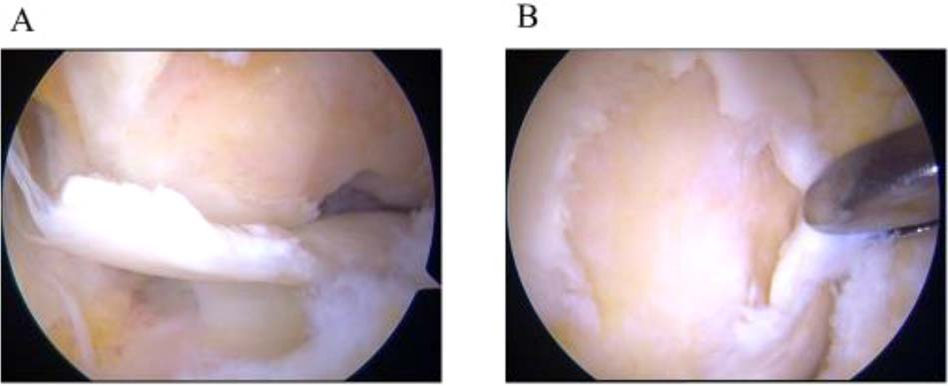
Arthroscopic findings. (a) Partially detached osteochondral fragment at the lateral femoral condyle. (b) Full-thickness cartilage defect after removal of the detached fragment.

**Figure 3:**
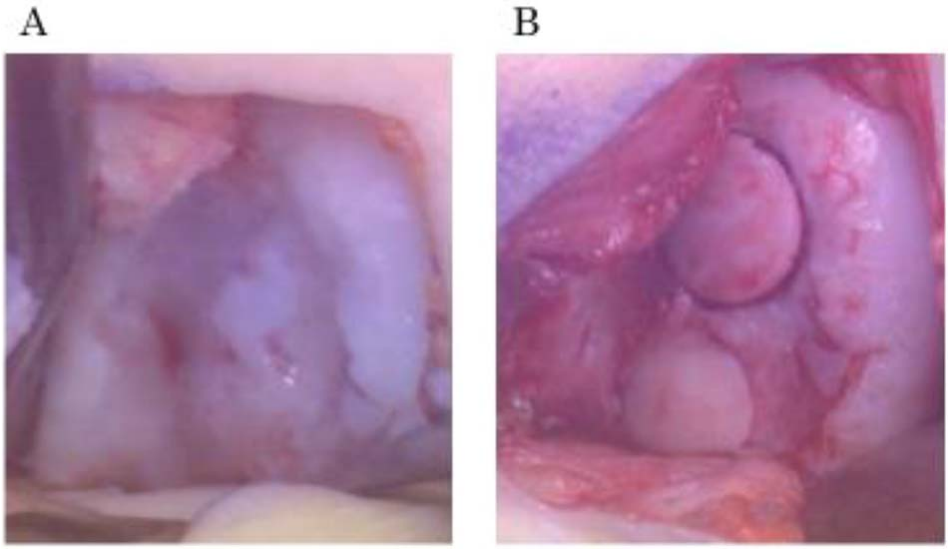
Intraoperative findings during OAT procedure. (a) Cartilage defect at the lateral condyle in the left knee. (b) Two osteochondral plugs transplanted into the recipient site.

A 31-year-old female with SLE, who had been receiving oral steroid therapy since the age of 16, presented with severe pain in her left knee without any particular trigger. Physical examination showed local swelling and effusion in the left knee, and the passive ranges of motion for both extension and flexion were limited to −30° and 130°, respectively. Ligamentous instability or apparent malalignment was not noted. Radiographic examination of the knee showed flattened contour of the lateral femoral condyle and irregularity of the articular surface (Fig. 1a). MRI showed osteonecrosis extending from the lateral femoral condyle to the femoral trochlea, whereas a tear of the lateral discoid meniscus was also identified (Fig. 1b and c). A sagittal fat-saturated T2-weighted image of the contralateral right knee also showed osteonecrosis lesion in the lateral condyle (Fig. 1d). Based on those clinical and image findings, diagnoses of steroid-induced osteonecrosis of the femoral condyle and a tear of the lateral discoid meniscus were made. An urgent arthroscopy was performed as the patient was experiencing severe knee pain and limited extension of the knee because of detached osteochondral fragments and discoid meniscal tear. First, the ganglion connected to the anterior horn of the lateral discoid meniscus was resected, and a partial meniscectomy (meniscoplasty) was performed. A 35 × 45 mm cartilage defect was found in the lateral femoral condyle, and the detached osteochondral fragments were removed (Fig. 2a and b). After the removal of detached chondral lesions under arthroscopy, the limitation of the ROM and severe pain was resolved. However, a large osteonecrosis lesion with osteochondral defects remained, which presumably lead to secondary osteoarthritis. Therefore, OAT was planned as a second-stage surgery. At the OAT procedure, the contralateral knee could not be used for graft harvest because of the presence of osteonecrosis in the femoral condyle. Therefore, only two 10-mm diameter osteochondral grafts could be harvested from the non-weight bearing area of the medial femoral condyle in the ipsilateral knee, and then inserted into the recipient site (Fig. 3a and b). Since only part of the lesion area could be replaced by the host tissue, microfracture was performed in the remaining lesion. Postoperatively, ROM exercise was started immediately, whereas weight bearing was not permitted for the initial 4 weeks and gradually returned to full weight thereafter.

**Figure 4:**
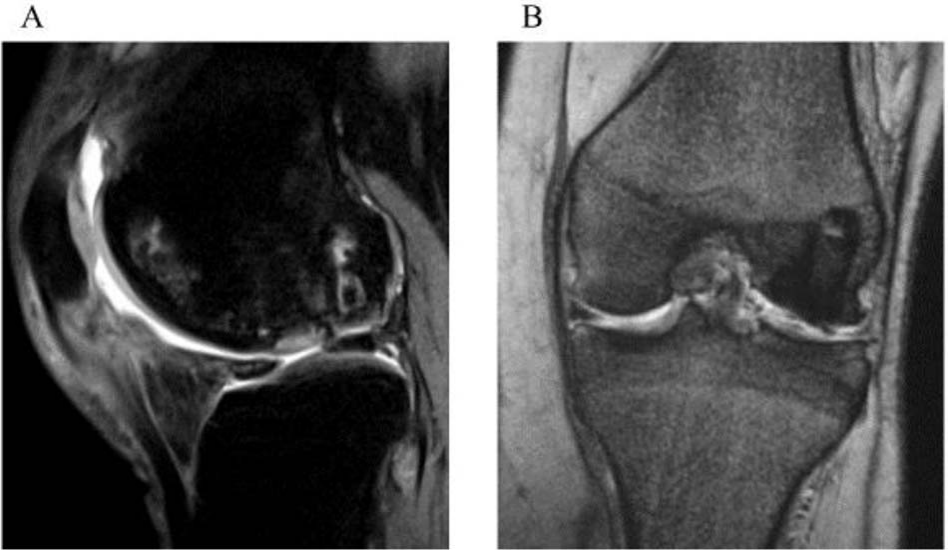
Two-year postoperative MRI. A sagittal fat saturation T2-weighted image (a) and a coronal T2-weighted image (b) show smooth articular surface in the grafted area and consolidation of the transplanted bone plugs.

One year after surgery, the visual analog scale (VAS) score had improved to 0, and the ROM for extension and flexion were recovered to 0° and 140°, respectively. At 2 years, the Knee Injury and Osteoarthritis Outcome Score improved to 494, the VAS score remained at 0, and ROM of extension and flexion were fully recovered to 0°–145°. The follow-up MRI exhibited healing and consolidation of the osteochondral graft with the smooth articular surface attained at the grafted area (Fig. 4a and b).

## Discussion

Secondary osteonecrosis caused by steroids has specific clinical features including multifocal or multiple lesions, extensive bony involvement, and deep to the subchondral region, presence of underlying morbidities [5–7]. Those associated factors affect the prognosis of steroid-induced osteonecrosis resulting in inferior treatment outcomes. As for the treatment of steroid-induced osteonecrosis of the knee, several options such as microfracture [8], OAT, bone grafting [9], knee osteotomy [10], Unicompartmental or Total Knee Arthroplasty [11] have been proposed; however, there is no consensus on optimal surgical option. Although microfracture is the minimally invasive surgery, repair tissue is composed of fibrous cartilage and its mechanical structural properties are considered inferior to those of normal hyaline cartilage [12]. In this case, taking the patient’s age, activity level, and risk of osteoarthritis into consideration, OAT was adopted as a surgical option. The advantage of OAT is the ability to replace pathological tissue by healthy bone and hyaline cartilage [4, 12]. In contrast, disadvantages of OAT are donor site morbidities and limited graft source [13]. Several studies reported satisfactory mid- to long-term follow-up results of OAT for osteonecrosis of the knee [2–4, 14, 15]. Graft donor site in OAT of the knee is generally peripheral region of the femoral trochlea in the ipsilateral knee; however, quantity of the graft can be insufficient to replace a large lesion. In the present case, the lesion was large, and the necrosis had extended to the condyles of bilateral knees. Therefore, the donor site was limited to the non-weight bearing area of the medial femoral condyle in the affected knee, which allowed us to harvest only two grafts with the diameter of 10 mm. Consequently, <20% of the entire lesion could be replaced with the osteochondral graft. As for the area that should be replaced by the graft in the whole lesion to attain satisfactory healing is controversial. However, MRI taken 2 years postoperatively showed the smooth articular surface of the grafted area and the patient did not report any pain or symptoms. In this report, the entire lesion area could not be repaired; however, favorable results could be attained at 2 years. The present case shows that only partial osteochondral grafting in the weight-bearing area may provide satisfactory results following OAT for a large steroid-induced osteonecrosis lesion of the knee.

## Data Availability

Not applicable.
